# Job Strain and the Risk of Inflammatory Bowel Diseases: Individual-Participant Meta-Analysis of 95 000 Men and Women

**DOI:** 10.1371/journal.pone.0088711

**Published:** 2014-02-18

**Authors:** Katriina Heikkilä, Ida E. H. Madsen, Solja T. Nyberg, Eleonor I. Fransson, Kirsi Ahola, Lars Alfredsson, Jakob B. Bjorner, Marianne Borritz, Hermann Burr, Nico Dragano, Jane E. Ferrie, Anders Knutsson, Markku Koskenvuo, Aki Koskinen, Martin L. Nielsen, Maria Nordin, Jan H. Pejtersen, Jaana Pentti, Reiner Rugulies, Tuula Oksanen, Martin J. Shipley, Sakari B. Suominen, Töres Theorell, Ari Väänänen, Jussi Vahtera, Marianna Virtanen, Hugo Westerlund, Peter J. M. Westerholm, G. David Batty, Archana Singh-Manoux, Mika Kivimäki, for the IPD-Work Consortium

**Affiliations:** 1 Finnish Institute of Occupational Health, Helsinki, Finland; 2 National Research Centre for the Working Environment, Copenhagen, Denmark; 3 Institute of Environmental Medicine, Karolinska Institutet, Stockholm, Sweden; 4 School of Health Sciences, Jönköping University, Jönköping, Sweden; 5 Stress Research Institute, Stockholm University, Stockholm, Sweden; 6 Department of Occupational and Environmental Medicine, Bispebjerg University Hospital, Copenhagen, Denmark; 7 Federal Institute for Occupational Safety and Health (BAuA), Berlin, Germany; 8 Institute for Medical Sociology, Medical Faculty, University of Düsseldorf, Düsseldorf, Germany,; 9 School of Community and Social Medicine, University of Bristol, Bristol, United Kingdom; 10 Department of Epidemiology and Public Health, University College London, London, United Kingdom; 11 Department of Health Sciences, Mid Sweden University, Sundsvall, Sweden; 12 Department of Public Health, University of Helsinki, Helsinki, Finland; 13 Department of Psychology, Umeå University, Umeå, Sweden; 14 The Danish National Centre for Social Research, Copenhagen, Denmark; 15 Finnish Institute of Occupational Health, Helsinki, Tampere and Turku, Finland; 16 Department of Public Health and Department of Psychology, University of Copenhagen, Copenhagen, Denmark; 17 Folkhälsan Research Center, Helsinki, Finland; 18 Department of Public Health, University of Turku, Turku, Finland; 19 Nordic School of Public Health, Göteborg, Sweden; 20 Occupational and Environmental Medicine, Uppsala University, Uppsala, Sweden; 21 Centre for Cognitive Ageing and Cognitive Epidemiology, University of Edinburgh, Edingurgh, United Kingdom; 22 Inserm U1018, Centre for Research in Epidemiology and Population Health, Villejuif, France; University of California, Los Angeles, United States of America

## Abstract

**Background and Aims:**

Many clinicians, patients and patient advocacy groups believe stress to have a causal role in inflammatory bowel diseases, such as Crohn's disease and ulcerative colitis. However, this is not corroborated by clear epidemiological research evidence. We investigated the association between work-related stress and incident Crohn's disease and ulcerative colitis using individual-level data from 95 000 European adults.

**Methods:**

We conducted individual-participant data meta-analyses in a set of pooled data from 11 prospective European studies. All studies are a part of the IPD-Work Consortium. Work-related psychosocial stress was operationalised as job strain (a combination of high demands and low control at work) and was self-reported at baseline. Crohn's disease and ulcerative colitis were ascertained from national hospitalisation and drug reimbursement registers. The associations between job strain and inflammatory bowel disease outcomes were modelled using Cox proportional hazards regression. The study-specific results were combined in random effects meta-analyses.

**Results:**

Of the 95 379 participants who were free of inflammatory bowel disease at baseline, 111 men and women developed Crohn's disease and 414 developed ulcerative colitis during follow-up. Job strain at baseline was not associated with incident Crohn's disease (multivariable-adjusted random effects hazard ratio: 0.83, 95% confidence interval: 0.48, 1.43) or ulcerative colitis (hazard ratio: 1.06, 95% CI: 0.76, 1.48). There was negligible heterogeneity among the study-specific associations.

**Conclusions:**

Our findings suggest that job strain, an indicator of work-related stress, is not a major risk factor for Crohn's disease or ulcerative colitis.

## Introduction

Inflammatory bowel diseases, Crohn's disease and ulcerative colitis, are incurable abnormalities of the adaptive mucosal immune response in the small and large intestines. Crohn's disease is perpetual inflammation of the small intestine, mainly driven by a sub-population of mucosal CD4+ T cells and T-helper type 1 (Th-1) cells, and marked by superficial and deep ulceration of the small intestine, with intestinal and perianal abscesses and fistulae [1]. Ulcerative colitis is mucosal inflammation of the rectum and colon, and is characterised by an abnormal T-helper type 2 (Th-2) response, which leads to epithelial cell cytotoxicity, increased apoptosis and epithelial barrier dysfunction in the colorectal area [2]. Typical symptoms of both inflammatory bowel diseases are abdominal pain, recurring diarrhoea, blood in faeces, fatigue and weight loss. Differential diagnosis between these diseases is usually made based on endoscopies or various types of imaging and scans.

Crohn's disease and ulcerative colitis are often diagnosed in individuals aged between 20 and 40 and whilst Crohn's disease is 20–30% more common in women, colitis occurs 60% more often in men [1]. Both diseases are rare at population level, as the prevalence of Crohn's disease in the Western countries is approximately 50 to 200 per 100 000 and of colitis about 120 to 200 per 100 000 [1]. Both also have a genetic background [3] and family history is indeed the most important predictor of developing either of them [4], [5]. Risk factors for Crohn's disease and ulcerative colitis include dietary factors (such as low fruit, vegetable and fibre intake), appendectomy, and medications [4], [6], [7]. Evidence from observational studies suggest that tobacco smoking is associated with an increased risk of Crohn's disease but a decreased risk of ulcerative colitis [4], which highlights the importance of investigating these diseases separately in risk factor studies. A further less well understood risk factor implicated in inflammatory bowel diseases is stress. It has been suggested that stress could initiate or reactivate gastrointestinal inflammation that causes the symptoms of inflammatory bowel diseases. [8]–[10] Stress activates neural pathways from the hypothalamus to the sympathetic and parasympathetic nervous systems, which in turn link to the enteric nervous system that controls gut motility and endocrine and exocrine functions in the gastrointestinal tract. [9], [11]

Many patients with Crohn's disease or ulcerative colitis believe stress to have caused or triggered their disease [10]. Epidemiological evidence from clinic-based studies, along with a recent systematic review pooling together findings from animal studies and observational studies in humans, indicated that stress can indeed worsen the symptoms and is associated with exacerbations in both diseases. [8], [12]–[14] Preclinical data also suggest that stress has a role in experimental colitis. [14] However, the observational epidemiological evidence for the associations between stress and the onset of Crohn's disease and ulcerative colitis is based on a small number of retrospective studies, which have had conflicting findings, with positive as well as null-results reported [9], [15], [16]. Findings from studies based on retrospectively collected data are prone to recall and other biases and cannot provide reliable evidence for an association between stress and the risk of disease.

We examined the hypothesis that stress is associated with risk of developing Crohn's disease and ulcerative colitis using prospectively collected data from 11 European studies with over 95 000 participants. As these diseases are most often diagnosed in working-age individuals, we focused on stress at work, operationalised as job strain, and the incidence of these diseases and conducted individual-participant data meta-analyses of job strain and the risk of Crohn's disease and ulcerative colitis.

## Methods

### Studies and participants

Individual-level data were obtained from the following 11 prospective cohort studies: Copenhagen Psychosocial Questionnaire I and II (COPSOQ-I and COPSOQ-II), Danish Work Environment Cohort Study (DWECS), Finnish Public Sector study (FPS), Health and Social Support (HeSSup), Intervention Project on Absence and Well-being (IPAW), Burnout, Motivation and Job Satisfaction study (Danish acronym PUMA), Still Working, Whitehall II and Work Lipids and Fibrinogen (WOLF) Norrland and Stockholm studies). These studies, begun between 1986 and 2005 in Finland, Sweden, Denmark and the UK, are a part of the “Individual-participant-data Meta-analysis in Working Populations” (IPD-Work) Consortium [17]. Each study in the IPD-Work consortium was approved by the relevant local or national ethics committees and all participants gave informed consent to take part. Details of the ethical approval are provided in Appendix S1. Details of the design and participants in all the studies have been published previously and are described, with references to previous publications, in Appendix S1. Our analyses were based on men and women who were employed at study baseline and had complete data on job strain, inflammatory bowel disease outcomes and potential confounders. Participants with a diagnosis of Crohn's disease (n = 108) or colitis (n = 271) before the study baseline or within the first month of follow-up were excluded from the analyses.

### Job strain exposure

Job strain, which is the most extensively used operationalisation of work stress, was ascertained from the baseline self-report questionnaire [18], [19]. Detailed descriptions of the job strain instrument and its harmonisation have been published previously [20]. Briefly, participants were asked to rate various psychosocial aspects of their job (statements such as “my job requires working very fast”) and mean response scores were calculated for job demands items and job control items for each participant. A job demands score higher than the study-specific median score was defined as “high demands” and a job control score lower than the study-specific median score as “low control”. Job strain was defined as high demands and low control at work.

### Inflammatory bowel disease outcomes

In each study, the participants' records were linked to national hospitalisation registries (including in-patient and out-patient appointments) and incident Crohn's disease and ulcerative colitis events during the study follow-up and the dates for these events were ascertained from these registers. In the Finnish studies (FPS, HeSSup and Still Working), we also used information from the Medical Reimbursement Register, kept by the Social Insurance Institution of Finland, to identify Crohn's disease and colitis events. All permanent residents in Finland are eligible for special reimbursement for the cost of medication for certain diseases, including Crohn's disease or colitis (75% of the cost during the study follow-up). A condition for eligibility is a diagnosis of Crohn's disease or ulcerative colitis made in specialised health care or by a specialist physician in gastroenterology, internal medicine, paediatrics or surgery, which are further reviewed by the expert board of the Social Insurance Institute. [21] The dates for the disease events ascertained from the Medical Reimbursement Register were the dates when the right to special reimbursement was granted.

Crohn's disease events were defined as diagnoses that were registered as International Classification of Diseases (ICD) version 10 code K50 and ICD-9 and earlier versions codes beginning with 555. Ulcerative colitis events were defined correspondingly as K51 and codes beginning with 556.

### Potential confounders

Potential confounders were identified based on a priori knowledge of their associations with job strain and Crohn's disease or ulcerative colitis. Potentially confounding factors were age and sex [1], [22], [23], socioeconomic position [6], [24], tobacco smoking [4], [25] and body mass index [7], [26]. There are a number of recognised as well as proposed risk factors to Crohn's disease and ulcerative colitis, including appendectomy, infections, dietary factors and medications [6], but there is currently no evidence of these being associated with job strain and our analyses were not adjusted for these.

Baseline data on all potential confounders were harmonised to be consistent across the studies. Participants' sex and age were ascertained from population registries or interview (in COPSOQ-I, COPSOQ-II, DWECS, FPS, IPAW, PUMA, Still Working, WOLF Norrland and WOLF Stockholm) or were self-reported (in HeSSup and Whitehall II). Socioeconomic position was defined based on occupational title, which was register-based (in COPSOQ-I, COPSOQ-II, DWECS, FPS, IPAW, PUMA and Still Working) or self-reported (in Whitehall II, WOLF Norrland and WOLF Stockholm). In HeSSup, socioeconomic position was based on self-reported highest educational qualification. Socioeconomic position was categorised into low, intermediate, high and other [26]. Tobacco smoking was ascertained from self-report and categorised into never, ex- and current smoking. Body mass index (BMI: weight in kilograms divided by height in meters squared) was calculated using height and weight, which were measured by baseline assessors (in Whitehall II, WOLF Norrland and WOLF Stockholm) or self-reported (in COPSOQ-II, DWECS, FPS, HeSSup, IPAW and PUMA). BMI data were not collected in COPSOQ-I and Still Working. BMI was categorised according to the World Health Organization recommendations into <18.5 kg/m^2^ (underweight), 18.5–24.9 kg/m^2^ (normal weight), 25–29.9 kg/m^2^ (overweight) and > = 30 kg/m^2^ (obese). Extreme BMI values (<15 or >50), which were probably due to measurement error, were excluded from the analysis.

### Statistical analyses

Job strain was modelled as a binary variable, job strain (high demands and low control) versus no strain (all other combinations of demands and control). Incident Crohn's disease and ulcerative colitis were binary outcomes. The associations between job strain and incident Crohn's disease or ulcerative colitis were modelled using Cox proportional hazards regression. Time at risk was defined as beginning at birth and observation time as starting at study baseline. The inflammatory bowel disease cases' follow-up ended at the date of their first disease event (Crohn's disease or ulcerative colitis). Participants who were free of these diseases were followed-up until their date of death or end of the hospital registry follow-up. The proportional hazards assumption was checked visually, by comparing the plots of log hazards against time in the exposed and the unexposed, and by using the Schoenfeld-test, and found valid. We ran age and sex-adjusted and multivariable- (age, sex, socioeconomic position, smoking and BMI)-adjusted models in each study in turn. As BMI was not measured in COPSOQ-I and Still Working, the multivariable-adjusted models in these studies were not adjusted for it. The study-specific effect estimates were pooled using fixed effect and random effects meta-analyses [27] and heterogeneity in the effect estimates was assessed using the I^2^ statistic. All statistical analyses were conducted using Stata 11 (Stata Corporation Ltd., College Station, Texas, US) apart from study-specific analyses in COPSOQ-I, COPSOQ-II, DWECS, IPAW and PUMA, which were conducted using SAS 9.2 (SAS Institute Inc., Cary, North Carolina, US).

## Results

Of the 95 379 participants who were free of Crohn's disease or ulcerative colitis at baseline, 126 men and women developed Crohn's disease and 414 developed ulcerative colitis during the follow-up. 94 839 participants remained free of these diseases throughout follow-up. COPSOQ-II, IPAW, PUMA, Still Working and WOLF Norrland were excluded from the analyses of job strain and Crohn's disease risk as no-one with job strain went on to develop Crohn's disease and these analyses were thus based on 111 incident cases of Crohn's disease (Table 1). Median follow-up was 10.5 years. Participant characteristics are provided in Appendix S2, Table S1. A sensitivity analysis showed that the exclusion of COPSOQ-II, IPAW, PUMA, Still Working and WOLF Norrland had not influenced our analyses of job strain and Crohn's disease risk (Appendix S2, Table S2).

**Table 1:** Job strain at baseline and inflammatory bowel diseases during the follow-up, by study.

Study, country	Baseline	Median (range) follow-up time (years)	N (%) free of Crohn's disease or ulcerative colitis	N (%) incident Crohn's disease	N (%) incident ulcerative colitis	N (%) job strain
Copenhagen Psychosocial Questionnaire I (COPSOQ-I), Denmark	1997	12.1 (0.02, 12.1)	1 706 (99.3)	4 (0.2)	8 (0.5)	354 (20.6)
Copenhagen Psychosocial Questionnaire II (COPSOQ-II), Denmark	2004–2005	5.0 (0.4, 5.3)	3 314 (99.79	1^1^ (0.03)	9 (0.3)	470 (14.1)
Danish Work Environment Cohort Study (DWECS), Denmark	2000	9.1 (0.1, 10.0)	5 394 (99.5)	5 (0.1)	20 (0.4)	1 203 (22.2)
Finnish Public Sector (FPS), Finland	2000	9.2 (0.06, 11.0)	43 683 (99.5)	52 (0.1)	158 (0.4)	7 044 (16.1)
Health and Social Support (HeSSup), Finland	1998	7.0 (0.04, 7.7)	14 909 (99.6)	14 (0.1)	41 (0.3)	2 642 (17.7)
Intervention Project on Absence and Well-being (IPAW), Denmark	1996–1997	13.0 (0.6, 13.79	1 949 (99.7)	2^1^ (0.1)	3 (0.2)	338 (17.3)
Burnout, Motivation and Job Satisfaction study (Danish acronym PUMA), Denmark	1999–2000	10.1 (0.5, 10.9)	1 747 (99.3)	5^1^ (0.3)	8 (0.5)	266 (15.1)
Still Working, Finland	1986	22.8 (0.03, 23.4)	8 979 (99.1)	15 (0.2)	66 (0.7)	1 412 (15.6)
Whitehall II, UK	2003–2004	6.5 (0.08, 7.3)	2 959 (98.4)	4 (0.1)	43 (1.4)	499 (16.6)
Work Lipids and Fibrinogen (WOLF) Norrland, Sweden	1996–1998	11.8 (0.1, 11.6)	4 637 (99.2)	7^1^ (0.1)	31 (0.7)	597 (12.8)
Work Lipids and Fibrinogen (WOLF) Stockholm, Sweden	1992–1995	14.8 (0.04, 18.2)	5 562 (99.2)	17 (0.3)	27 (0.5)	903 (16.1)
**All**	**1986–2005**	**10.5 (0.02, 23.4)**	**94 839 (99.4)**	**126 (0.1)**	**414 (0.4)**	**15 728 (16.5)**
				**111 (0.1)^2^**		

1 COPSOQ-II, IPAW, PUMA and WOLF Norrland were excluded from the analyses of job strain and Crohn's disease risk because in these studies no-one with job strain developed Crohn's disease.

2 After exclusions NB: Inflammatory bowel diseases here refer to Crohn's disease and ulcerative colitis.

**Figure 1:**
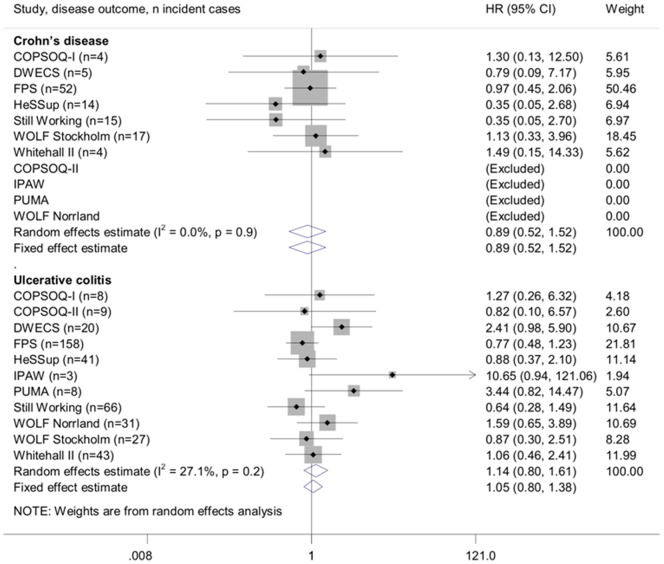
Age and sex-adjusted associations of job strain with Crohn's disease or ulcerative colitis.

**Figure 2:**
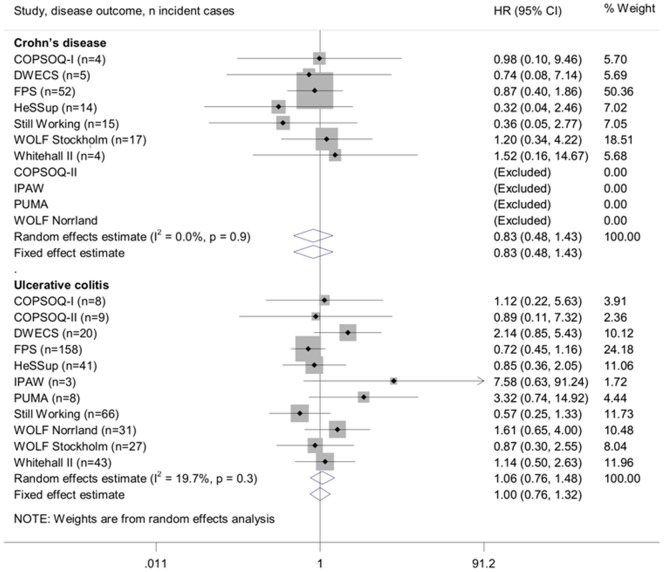
Multivariable-adjusted associations of job strain with Crohn's disease or ulcerative colitis (adjusted for age, sex, socioeconomic position, smoking and BMI).

The age and sex-adjusted associations between job strain and incident Crohn's disease and ulcerative colitis are shown in Figure 1 and multivariable-adjusted associations in Figure 2. Job strain was not associated with the risk of developing Crohn's disease in the age and sex-adjusted analyses (random effects hazard ratio (HR): 0.89, 95% CI: 0.52, 1.52) Further adjustment (in addition to age and sex) for socioeconomic position, BMI and smoking did not markedly change this estimate (random effects HR: 0.83, 95% CI: 0.48, 1.43). There was little heterogeneity in the estimated associations between job strain and incident Crohn's disease (I^2^: 0%).

The association estimates for ulcerative colitis were more heterogeneous than the estimates for Crohn's disease (with approximately 20–30% heterogeneity in minimum- and multivariable-adjusted analyses). Job strain was not associated with the risk of ulcerative colitis in the age and sex-adjusted analyses (random effects HR: 1.14, 95% CI: 0.80, 1.61). Further adjustment for socioeconomic position, BMI and smoking attenuated this estimate even closer to the null-value (random effects HR: 1.06, 95% CI: 0.76, 1.48).

## Discussion

### Summary of findings in the context of previous research

Better understanding the association between work-related stress and the risk of inflammatory bowel diseases is important because both Crohn's disease and ulcerative colitis are often diagnosed in working-age individuals [1]. Our meta-analyses suggest that job strain, the most extensively studied operationalisation of work-related psychosocial stress, is not associated with the risk of Crohn's disease or ulcerative colitis after adjustment for age, sex, socioeconomic position, smoking and BMI, thus providing evidence against the belief shared by many patients that work-related stress might have a causal role in their disease [10]. These findings do not, of course, take away from the evidence from cross-sectional studies that inflammatory bowel disease patients often experience stress [28], [29], or that stress can trigger symptoms and exacerbations in these diseases [8]. The trigger-effect is evident in studies in which the time from the stressful event or experience to the onset of symptoms or diagnosis of inflammatory bowel disease is relatively short. Our analyses, however, were based on several years of follow-up, which is ideal for disentangling the possible impact of job strain on the disease process. Also, our findings are not necessarily generalisable to other indicators of work-related stress or sources of stress outside work. Further large, prospective, population-based studies would help to determine whether other operationalisations of work-related stress or stress in other areas of life have a role in the aetiology of inflammatory bowel diseases

### Strengths and limitations

The main strength of the IPD-Work Consortium is its size, which allowed us to investigate relatively rare diseases, such as Crohn's disease and colitis, in a prospective cohort design. Thus far the evidence for an association between stress and the risk of inflammatory bowel diseases has been based on small, retrospective studies, with findings prone to recall and other biases [9], [15], [16]. The lack of large, prospective, population-based studies may relate, at least partly, to inflammatory bowel diseases being rare at population-level (the incidence of Crohn's disease in the Western countries is about 6 to 17 per 100 000 and of colitis about 8 to 17 per 100 000).[1] The need to recruit large numbers of participants to ensure sufficient statistical power for the analyses is evident in our investigation which, as far as we are aware, is the largest study of this topic thus far yet still includes just over 100 incident cases of Crohn's disease and 400 incident cases of colitis. The incidence of both inflammatory bowel diseases was lower among the men and women included in our analyses than in the general population, which we suspect is because one eligibility criterion for our analyses was that the participants had to work at study baseline and the working population tends to be healthier than those who do not work.

A further strength in our analyses is that incident Crohn's disease and colitis cases were ascertained from hospitalisation and drug reimbursement registries, which generally have good coverage and are not prone to recall bias. Inflammatory bowel disease diagnoses are made by specialist clinicians using endoscopies or imaging. These techniques allow differential diagnosis of Crohn's and colitis, which is a further strength of our study. The diagnostic tests can be done in primary health care where facilities exist, or at out-patient hospital appointments, but even those initially diagnosed in primary care attend hospital early on in the disease process, for confirmation of their diagnosis or planning and monitoring of their treatment. The hospitalisation data in the studies analysed here covered outpatient appointments onwards from the mid-1990s in the Danish and Finnish studies [30] and from 2003 in the UK study [31]. The Swedish data on specialised outpatient care were available from 2001 and have been shown to have good coverage from 2005-06 onwards [32]. Although there have been some concerns about its previous coverage, the validity of the diagnoses is likely to be good. Thus, as we used registers to ascertain incident cases, it is possible that a small number of individuals who had an inflammatory bowel disease before the study baseline were misclassified as disease-free at the start of the follow-up and defined as incident cases according to a later hospitalisation or drug reimbursement record. Also, misdiagnoses can occur and it is possible that as a result, some healthy individuals have been misclassified as having Crohn's disease or ulcerative colitis, or individuals with either of these diseases misclassified as healthy or having the incorrect disease. Such misclassification is unlikely to be related to any job strain exposure and thus it may have diluted our association estimates, though probably not substantially.

Generally, our analyses of prospectively collected data are unlikely to have been influenced by reverse causality. However, as the symptoms of inflammatory bowel diseases can be vague and differential diagnosis, with the various tests required, can take time. It is therefore possible that the symptoms of an underlying disease have contributed to some individuals reporting high job demands and (though less likely) low control at baseline. If anything, this should artificially inflate the estimated associations between job strain and inflammatory bowel diseases, a result that was not seen in the present analyses Also, the follow-up in the studies included in our analyses span two decades, during which the diagnostic methods for inflammatory bowel disease have changed and improved. It is possible that some outcome misclassification (e.g. irritable bowel syndrome misdiagnosed as inflammatory bowel disease) may have occurred, especially during the early years of follow-up in the older studies. Due to the rarity of inflammatory bowel diseases and the relatively small numbers of cases in our analyses, we were unable to conduct sensitivity analyses excluding cases diagnosed during the early follow-up, though individuals with an inflammatory bowel disease diagnosis within the very first month of follow-up were left out of the analyses. We used a well-recognised, validated and harmonised exposure measure, job strain [18], [20]. However, we did not have data on biological markers of stress, such as dysregulation of the hypothalamic-pituitary-adrenal axis or the activation of the autonomous nervous system, and were thus unable to explore the potential biological pathways linking stress and inflammatory bowel diseases.

## Conclusions

Our findings suggest that job strain is unlikely to be an important risk factor for developing Crohn's disease or ulcerative colitis. Whilst these findings would not change the current clinical practice, they provide information to alleviate concerns by patients that work-related stress, as indicated by job strain, would lead to either disease. Further large, prospective, population-based studies would help to determine whether other types of work-related stress or stress in other areas of life have a role in the aetiology of inflammatory bowel diseases.

## Supporting Information

Appendix S1 **Individual-participant Data Meta-analysis of Working Populations (IPD-Work) Consortium, studies and participants.**
(DOC)

Appendix S2 **Participant characteristics and a sensitivity analysis.**
(DOCX)

Checklist S1 **STROBE Checklist.**
(DOC)

Checklist S2 **MOOSE Checklist.**
(DOC)
