# Supplementary material for: Job Strain and the Risk of Inflammatory Bowel Diseases: Individual-Participant Meta-Analysis of 95 000 Men and Women
Source: PLoS One. 2014 Feb 18;9(2):e88711. doi: 10.1371/journal.pone.0088711 (PMC3928274; doi:10.1371/journal.pone.0088711)
Supplement: Appendix S1 — Individual-participant Data Meta-analysis of Working Populations (IPD-Work) Consortium, studies and participants. (DOC) [file pone.0088711.s001.doc]

**Appendix S1. Individual-participant Data Meta-analysis of Working Populations (IPD-Work) Consortium, studies and participants**

**Individual-participant Data Meta-analysis of Working Populations (IPD-Work) Consortium**

IPD-Work Consortium is a collaborative meta-analysis project that was established at the Four Centers Initiative Meeting (a meeting of stress researchers from University College London, the Institut National de la Santé et de la Recherche in Paris, the University of Dusseldorf in Germany, and Karolinska Institutet in Stockholm) in London in 2008. IPD-Work Consortium consists of 19 prospective European cohort studies. The overarching aim of the consortium is to investigate the effect of work-related stress on chronic diseases using data from studies with a measure of work-related stress at baseline and register-based information on incident chronic diseases during follow-up [1-5] .

Of the 19 studies, 11 studies were included in our meta-analyses and are described below. Six studies were excluded from the current analyses because no register-based outcome data on inflammatory bowel diseases were available (Belstress, GAZEL, Heinz-Nixdorf Recall study, Cooperative Health Research in the Region Augsburg (KORA) studies, German Socioeconomic Panel Study (German acronym SOEP) and Netherlands Working Conditions Survey (NWCS)). Further two studies were excluded because the numbers of incident inflammatory bowel disease events were too small to analyse (Swedish Longitudinal Occupational Survey of Health (SLOSH) and Permanent Onderzoek Leefsituatie (POLS)). It is unlikely that these reasons for exclusion would be associated with the study results or introduce notable bias to our meta-analyses. All studies provided individual-level data or conducted study-specific analyses according to our instructions and provided us with aggregate results. No study team refused to provide data or results.

**Studies and participants**

*Copenhagen Psychosocial Questionnaire version I (COPSOQ-I)*

COPSOQ-I is a prospective cohort study of a random sample of Danish residents selected from the Danish population register. The participants were 20-60 years of age at study baseline in 1997. A baseline questionnaire, information about the study and its aims and an invitation to take part was posted to 4 000 people and 2 454 individuals agreed to participate, of whom 1 858 were employed [6]. In Denmark, questionnaire- and register-based studies do not require approval from the Danish National Committee on Biomedical Research Ethics (Den Centrale Videnskabetiske komité). COPSOQ-I was approved by and registered with the Danish Data protection agency (registration number: 2008 - 54 - 0553). Responding to the baseline questionnaire was taken to imply informed consent to take part.

*Copenhagen Psychosocial Questionnaire version II (COPSOQ-II)*

COPSOQ-II was carried out in 2004-2005. It included a follow up of respondents from COPSOQ I and also a representative sample of Danish residents aged 20-60. The questionnaire, with information about the study, was sent to 8 000 individuals from the random sample and 4 732 individuals responded. The questionnaire could be completed using the posted questionnaire or via internet [7]. Of the 3 818 employed participants, 3 428 individuals had data on job strain and were eligible for our meta-analyses. In Denmark, questionnaire- and register-based studies do not require ethics committee approval. COPSOQ-II was approved by and registered with the Danish Data protection agency (registration number: 2004-54-1493). Responding to the baseline questionnaire was taken to imply informed consent to take part.

*Danish Work Environment Cohort Study (DWECS)*

DWECS is a split panel survey of working age Danish people. The cohort was established in 1990, when a simple random sample of men and women, aged 18-59, was drawn from the Danish population register. The participants have been followed up at five year intervals and data from the year 2000 was used for the IPD-Work. That year 11 437 individuals were invited to participate and 8 583 agreed to do so [8,9]. Of those, 5 606 individuals were employed. In Denmark, questionnaire- and register-based studies do not require ethics committee approval. DWECS was approved by and registered with the Danish Data protection agency (registration number: 2007-54-0059). Participants were provided information about the study with the baseline questionnaire and responding was taken to imply informed consent to take part.

*Finnish Public Sector study (FPS)*

The Finnish Public Sector study is a prospective cohort study comprising the entire public sector personnel of 10 towns (municipalities) and 21 hospitals in the same geographical areas. Participants, recruited from employers' records in 2000-2002, were individuals who were employed in the study organisations at the time of the questionnaire survey [10]. 48 592 individuals (9 337 men and 39 255 women aged 17 to 65) responded to the questionnaire. Ethical approval was obtained from the ethics committees of the Finnish Institute of Occupational Health and Helsinki and Uusimaa Hospital District. According to the Finnish law, written consent is not required for survey and register-based research, as long as that participation is voluntary, and the participants have been informed about the aims of the study and the possible register linkages [11]. Thus, responding to the questionnaire voluntarily (having had access to information on the study aims and possible register linkages) was taken to imply written consent.

*Health and Social Support (HeSSup)*

The Health and Social Support (HeSSup) study is a prospective cohort study of a stratified random sample of the Finnish population in the following four age groups: 20–24, 30–34, 40–44, and 50–54. The participants were identified from the Finnish population register and posted an invitation to participate, along with a baseline questionnaire, in 1998 [12]. Of the 25 898 respondents in 1998, 17 102 were employed. All participants gave written informed consent to take part.

*Intervention Project on Absence and Well-being (IPAW)*

IPAW is a 5-year psychosocial work environment intervention study including 22 intervention and 30 control work places in three organisations (a large pharmaceutical company, municipal technical services and municipal nursing homes) in Copenhagen, Denmark [13,14]. The baseline questionnaire was posted to all the employees at the selected work-sites between 1996 and 1997. Of the 2 721 employees who worked at the 52 IPAW sites, 2 068 men and women completed the baseline questionnaire. Interventions took place at 22 workplaces during 1996-98 at the organisational and interpersonal level. IPAW was approved by and registered with the Danish Data Protection Agency (registration number: 2000-54-0066). Participants were provided information about the study and with the baseline questionnaire and responding was taken to imply informed consent to take part.

*Burnout, Motivation and Job Satisfaction study (Danish acronym: PUMA)*

Burnout, Motivation and Job Satisfaction study (Danish acronym: PUMA) is an intervention study of burn-out among employees in the human service sector [15] . Selection criteria for the participating organisations was that they had between 200 and 500 employees, that occupational groups within each organisation were willing to participate and that the organisations would commit to the entire five-year study period. Participants gave consent to having their national identity numbers collected and used in later record linkages to Danish hospitalisation and cause of death registries (Hospitalsindlæggelsesregisteret, Dødsårsagsregisteret. At study baseline in 1999-2000, 1 914 participants agreed to take part. PUMA was approved by the Scientific Ethical Committees (Videnskabsetisk Komiteer) in the counties in which the study was conducted and approved by and registered with the Danish Data Protection Agency (registration number: 2000-54-0048).

*Still Working*

Still Working is an ongoing prospective cohort study. In 1986, the employees (n = 12 173) at all Finnish centres of operation of Enso Gutzeit (a forestry products manufacturer) were invited to participate in a questionnaire survey on demographic, psychosocial and health-related factors [16,17] and 9 282 individuals participated. The study was approved by the ethics committee of the Finnish Institute of Occupational Health.

*Whitehall II*

The Whitehall II study is a prospective cohort study set up to investigate socioeconomic determinants of health. At study baseline in 1985-1988, 10 308 civil service employees (6 895 men and 3 413 women) aged 35-55 and working in 20 civil service departments in London were invited to participate in the study [18]. The Whitehall II study protocol was approved by the University College London Medical School committee on the ethics of human research. Written informed consent was obtained at each data collection wave.

*WOLF (Work, Lipids, and Fibrinogen) Norrland and Stockholm studies*

The WOLF (Work, Lipids, and Fibrinogen) Stockholm study is a prospective cohort study of 5 698 people (3 239 men and 2 459 women) aged 19–70 and working in companies in Stockholm county [19]. WOLF Norrland is a prospective cohort of 4 718 participants aged 19-65 working in companies in Jämtland and Västernorrland counties [20]. At study baseline the participants underwent a clinical examination and completed a set of health questionnaires. For WOLF Stockholm, the baseline assessment was undertaken at 20 occupational health units between November 1992 and June 1995 and for WOLF Norrland at 13 occupational health service units in 1996-98. The Regional Research Ethics Board in Stockholm, and the ethics committee at Karolinska Institutet, Stockholm, Sweden approved the study. The participants received written and verbal information about the study and participation was voluntary. Answering the baseline questionnaire was taken to imply informed consent to participate.

**References**

1. Fransson EI, Heikkilä K, Nyberg ST, Zins M, Westerlund H, et al. (2012) Job strain as a Risk Factor for Sedentary Lifestyle: An Individual-Participant Meta-analysis of up to 170 000 Men and Women. The IPD-Work Consortium. American Journal of Epidemiology In press.

2. Kivimaki M, Nyberg ST, Batty GD, Fransson E, Heikkila K, et al. (2012) Job strain as a risk factor for future coronary heart disease: Collaborative meta-analysis of 2358 events in 197,473 men and women. Lancet 380: 1491-1497.

3. Heikkila K, Nyberg ST, Fransson EI, Alfredsson L, De Bacquer D, et al. (2012) Job Strain and Alcohol Intake: A Collaborative Meta-analysis of Individual-participant Data from 140 000 Men and Women. PLoS ONE 7: e40101.

4. Heikkilä K, Nyberg ST, Fransson EI, Alfredsson L, De Bacquer D, et al. (2012) Job Strain and Tobacco Smoking: An Individual-participant Data Meta-analysis of 166 130 Adults in 15 European Studies. PLoS ONE 7: e35463.

5. Nyberg ST, Heikkila K, Fransson EI, Alfredsson L, De Bacquer D, et al. (2012) Job strain in relation to body mass index: pooled analysis of 160 000 adults from 13 cohort studies. Journal of Internal Medicine 272: 65-73.

6. Kristensen TS, Hannerz H, Hogh A, Borg V (2005) The Copenhagen Psychosocial Questionnaire--a tool for the assessment and improvement of the psychosocial work environment. Scand J Work Environ Health 31: 438-449.

7. Pejtersen JH, Kristensen TS, Borg V, Bjorner JB (2010) The second version of the Copenhagen Psychosocial Questionnaire. Scand J Public Health 38: 8-24.

8. Burr H, Bjorner JB, Kristensen TS, Tüchsen F, Bach E (2003) Trends in the Danish work environment in 1990–2000 and their associations with labor-force changes. Scand J Work Environ Health 29: 270-279.

9. Feveile H, Olsen O, Burr H, Bach E (2007) Danish Work Environment Cohort Study 2005: From idea to sampling design. Statistics in Transition 8: 441-458.

10. Kivimaki M, Lawlor DA, Smith GD, Kouvonen A, Virtanen M, et al. (2007) Socioeconomic Position, Co-Occurrence of Behavior-Related Risk Factors, and Coronary Heart Disease: the Finnish Public Sector Study. Am J Public Health 97: 874-879.

11. Justice FMo (1999) Personal Data Act (523/1999).

12. Korkeila K, Suominen S, Ahvenainen J, Ojanlatva A, Rautava P, et al. (2001) Non-response and related factors in a nation-wide health survey. Eur J Epidemiol 17: 991-999.

13. Nielsen M, Kristensen T, Smith-Hansen L (2002) The Intervention Project on Absence and Well-being (IPAW): design and results from the baseline of a 5-year study. Work and Stress 16: 191-206.

14. Nielsen ML, Rugulies R, Christensen KB, Smith-Hansen L, Bjorner JB, et al. (2004) Impact of the psychosocial work environment on registered absence from work: a two-year longitudinal study using the IPAW cohort. Work & Stress 18: 323-335.

15. Borritz M, Rugulies R, Bjorner JB, Villadsen E, Mikkelsen OA, et al. (2006) Burnout among employees in human service work: design and baseline findings of the PUMA study. Scand J Public Health 34: 49-58.

16. Kalimo R, Toppinen S (1999) Organizational well-being: ten years of research and development: in a forest industry corporation. In: Kompier M, Cooper C, editors. Preventing Stress, Improving Productivity: European Case Studies in the Workplace. London: Routledge. pp. 52-85.

17. Vaananen A, Murray M, Koskinen A, Vahtera J, Kouvonen A, et al. (2009) Engagement in cultural activities and cause-specific mortality: prospective cohort study. Prev Med 49: 142-147.

18. Marmot MG, Smith GD, Stansfeld S, Patel C, North F, et al. (1991) Health inequalities among British civil servants: the Whitehall II study. Lancet 337: 1387-1393.

19. Peter R, Alfredsson L, Hammar N, Siegrist J, Theorell T, et al. (1998) High effort, low reward, and cardiovascular risk factors in employed Swedish men and women: baseline results from the WOLF Study. J Epidemiol Community Health 52: 540-547

20. Alfredsson L, Hammar N, Fransson E, de Faire U, Hallqvist J, et al. (2002) Job strain and major risk factors for coronary heart disease among employed males and females in a Swedish study on work, lipids and fibrinogen. Scand J Work Environ Health 28: 238-248.
