# Supplementary material for: Job Strain and the Risk of Inflammatory Bowel Diseases: Individual-Participant Meta-Analysis of 95 000 Men and Women
Source: PLoS One. 2014 Feb 18;9(2):e88711. doi: 10.1371/journal.pone.0088711 (PMC3928274; doi:10.1371/journal.pone.0088711)
Supplement: Appendix S2 — Participant characteristics and a sensitivity analysis. (DOCX) [file pone.0088711.s002.docx]

**Appendix S2. Participant characteristics and a sensitivity analysis**

**Table S1. Participant characteristics**

| **Study, country** | **Baseline** | **N participants** | **N (%) women** | **Baseline characteristics** | | | |
| --- | --- | --- | --- | --- | --- | --- | --- |
|  |  |  |  | **Mean (SD) age** | **N (%) BMI**  <18.5  18.5-24.9  25-29.9  >=30 | **N (%) SEP**  Low  Medium  High  Other | **N (%) smokers**  Never  Ex-  Current |
| Copenhagen Psychosocial Questionnaire I (COPSOQ-I), Denmark | 1997 | 1 718 | 828 (48.2) | 40.9 (10.6) | Not measured. | 757 (44.1)  487 (28.4)  474 (27.6)  - | 666 (38.8)  421 (24.5)  631 (36.7) |
| Copenhagen Psychosocial Questionnaire II (COPSOQ-II), Denmark | 2004-2005 | 3 324 | 1 729 (52.0) | 42.7 (10.2) | 48 (1.4)  1786 (53.7)  1102 (33.2)  388 (11.7) | 1408 (42.4)  946 (28.5)  970 (29.2)  - | 1396 (42.0)  863 (26.0)  1 065 (32.0) |
| Danish Work Environment Cohort Study (DWECS), Denmark | 2000 | 5 419 | 2 518 (46.5) | 41.8 (11.0) | 91 (1.7)  3141 (58.0)  1768 (32.6)  419 (7.7) | 2 350 (43.4)  1642 (30.3)  1427 (26.3)  - | 2167 (40.0)  1244 (23.0)  2 008 (37.1) |
| Finnish Public Sector (FPS), Finland | 2000 | 43 893 | 35 407 (80.7) | 44.6 (9.5) | 533 (1.2)  24 419 (55.6)  13 987 (31.9)  4 954 (11.3) | 7 871 (17.9)  22 807(52.0)  12 753 (29.1)  462 (1.1) | 19 041 (43.4)  17 002 (38.7)  7 850(17.9) |
| Health and Social Support (HeSSup), Finland | 1998 | 14 964 | 8 438 (56.4) | 39.6 (10.3) | 232 (1.6)  8 396 (56.1)  4 861 (32.5)  1 475 (9.9) | 3 814 (25.5)  8 753 (58.5)  2 321 (15.5)  76 (0.5) | 6 610 (44.2)  4 434 (29.6)  3 920 (26.2) |
| Intervention Project on Absence and Well-being (IPAW), Denmark | 1996-1997 | 1 954 | 1 299 (66.5) | 41.2 (10.5) | 57 (2.9)  1178 (6.3)  555 (28.4)  164 (8.4) | 1317 (67.4)  278 (14.2)  359 (18.4)  - | 585 (29.9)  446 (22.8)  923 (47.2) |
| Burnout, Motivation and Job Satisfaction study (Danish acronym PUMA), Denmark | 1999-2000 | 1 760 | 1 446 (82.2) | 42.6 (10.3) | 35 (2,0)  1070 (60.8)  495 (28.1)  160 (9.1) | 747 (42.4)  516 (29.3)  497 (28.2)  - | 634 (36.0)  434 (24.7)  692 (39.3) |

**Table S1, continued. Participant characteristics**

| Still Working, Finland | 1986 | 9 060 | 2 068 (22.8) | 40.9 (9.1) | Not measured | 6 258 (69.1)  2 153 (23.8)  649 (7.2)  - | 3 186 (35.2)  2 884 (31.8)  2 990 (33.0) |
| --- | --- | --- | --- | --- | --- | --- | --- |
| Whitehall II, UK | 2003 - 2004 | 3 006 | 734 (24.4) | 56.7 (4.1) | 19 (0.6)  1 037 (34.5)  1 373 (45.7)  577 (19.2) | 224 (7.5)  1 285 (42.8)  1 497 (49.8)  - | 1 523 (50.7)  1 222 (40.7)  261 (8.7) |
| Work Lipids and Fibrinogen (WOLF) Norrland, Sweden | 1996–1998 | 4 675 | 769 (16.5) | 44.1 (10.3) | 14 (0.3)  1 881 (40.2)  2 153 (46.1)  627 (13.4) | 2 788 (59.6)  1 520 (32.5)  362 (7.7)  5 (0.1) | 2 361 (50.5)  1 431 (30.6)  883 (18.9) |
| Work Lipids and Fibrinogen (WOLF) Stockholm, Sweden | 1992–1995 | 5 606 | 2 417 (43.1) | 41.5 (11.0) | 81 (1.4)  3 250 (58.0)  1 845 (32.9)  430 (7.7) | 1 690 (30.2)  2 905 (51.8)  894 (16.0)  117 (2.1) | 2 636 (47.0)  1 555 (27.7)  1 415 (25.2) |
| **All** | **1986-2005** | **95 379** | **57 653 (60.4)** | **43.2** | 1 110 (1.3)  46 158 (54.6)  28 139 (33.3)  9 194 (10.9)* | 29 224 (30.6)  43 292 (45.4)  22 203 (23.3)  660 (0.7) | 40 805 (42.8)  31 936 (33.5)  22 638 (23.7) |

* The total in this column does not add up to 95 372 as BMI was not measured in two studies.

**Sensitivity analysis**

To investigate the possibility that to investigate the possibility that we had over-estimated the associations between job strain and Crohn’s disease by excluding from our main analyses the studies in which no-one with job strain had Crohn’s disease, we conducted sensitivity analyses in two sets of pooled individual level data. The first set contained data from FPS, HeSSup, Still Working, Whitehall II, WOLF Norrland and WOLF Stockholm. The second set contained data from COPSOQ-I, COPSOQ-II, DWECS, IPAW and PUMA. These analyses were conducted separately because the analyses in the Danish data cannot be extracted from the Statistics Denmark server for combining of the datasets.

We ran age and sex- and multivariable-adjusted Cox models in the pooled dataset, stratified by study. Age and sex-adjusted association estimates and multivariable-adjusted estimates were similar to those of our main analyses and are shown in Table S2.

Table S2. Associations between job strain and incident Crohn’s disease in pooled individual-level datasets

| **Pooled dataset** | **N (%) disease-free** | **N (%) Crohn’s disease** | **Age and sex-adjusted HR (95% CI)** | **Multivariable-adjusted HR (95% CI)** |
| --- | --- | --- | --- | --- |
| **Set 1:** FPS, HeSSup, Still Working, Whitehall II , WOLF Norrland and WOLF Stockholm | 80 729 (99.9) | 109 (0.1) | 0.78 (0.44, 1.36) | 0.82 (0.45, 1.48) |
| **Set 2:** COPSOQ-I, COPSOQ-II, DWECS, IPAW and PUMA | 14 110 (99.9) | 17 (0.1) | 0.58 (0.13, 2.54) | 0.50 (0.11, 2.51) |
