# Supplementary material for: Job Strain and the Risk of Inflammatory Bowel Diseases: Individual-Participant Meta-Analysis of 95 000 Men and Women
Source: PLoS One. 2014 Feb 18;9(2):e88711. doi: 10.1371/journal.pone.0088711 (PMC3928274; doi:10.1371/journal.pone.0088711)
Supplement: Checklist S2 — MOOSE Checklist. (DOC) [file pone.0088711.s004.doc]

**MOOSE Checklist**

**Article details**

**Title:** Job Strain and the Risk of Inflammatory Bowel Diseases: Individual-participant Meta-analysis of 95 000 Men and Women

**Short title:** Job Strain and Inflammatory Bowel Diseases

**Authors and author affiliations:** Katriina Heikkilä^1^, Ida E.H. Madsen^2^, Solja T. Nyberg^1^, Eleonor I. Fransson^3^ ^4,5^, Kirsi Ahola^1^, Lars Alfredsson^3^, Jakob B. Bjorner^2^, Marianne Borritz^6^, Hermann Burr^7^, Nico Dragano^8^, Jane E. Ferrie^9,10^, Anders Knutsson^11^, Markku Koskenvuo^12^, Aki Koskinen^1^, Martin L. Nielsen^6^, Maria Nordin^13^, Jan H. Pejtersen^14^, Jaana Pentti^15^, Reiner Rugulies^2,16^, Tuula Oksanen^15^, Martin J. Shipley^10^, Sakari B. Suominen^17-19^, Töres Theorell^5^, Ari Väänänen^1^, Jussi Vahtera^15^ ^18^, Marianna Virtanen^1^, Hugo Westerlund^5^, Peter J.M. Westerholm^20^, G. David Batty ^10, 21^, Archana Singh-Manoux^10,22^ and Mika Kivimäki^10^ ^1^; for the IPD-Work Consortium

1 Finnish Institute of Occupational Health, Helsinki, Finland.

2 National Research Centre for the Working Environment, Copenhagen, Denmark.

3 Institute of Environmental Medicine, Karolinska Institutet, Stockholm, Sweden.

4 School of Health Sciences, Jönköping University, Jönköping, Sweden.

5 Stress Research Institute, Stockholm University, Stockholm, Sweden.

6 Department of Occupational and Environmental Medicine, Bispebjerg University Hospital, Copenhagen, Denmark

7 Federal Institute for Occupational Safety and Health (BAuA), Berlin, Germany.

8 Institute for Medical Sociology, Medical Faculty, University of Düsseldorf, Düsseldorf, Germany.

9 School of Community and Social Medicine, University of Bristol, Bristol, UK.

10 Department of Epidemiology and Public Health, University College London, London, UK.

11 Department of Health Sciences, Mid Sweden University, Sundsvall, Sweden.

12 Department of Public Health, University of Helsinki, Helsinki, Finland.

13 Department of Psychology, Umeå University, Umeå, Sweden.

14 The Danish National Centre for Social Research, Copenhagen, Denmark.

15 Finnish Institute of Occupational Health, Helsinki, Tampere and Turku, Finland.

16 Department of Public Health and Department of Psychology, University of Copenhagen, Copenhagen, Denmark.

17 Folkhälsan Research Center, Helsinki, Finland.

18 Department of Public Health, University of Turku, Turku, Finland

19 Nordic School of Public Health, Göteborg, Sweden.

20 Occupational and Environmental Medicine, Uppsala University, Uppsala, Sweden.

21 Centre for Cognitive Ageing and Cognitive Epidemiology, University of Edinburgh.

22 Inserm U1018, Centre for Research in Epidemiology and Population Health, Villejuif, France.

| **Criteria** | | **Brief description of how the criteria were handled in the meta-analysis** |
| --- | --- | --- |
| **Reporting of background should include** | |  |
| √ | Problem definition | Introduction |
| √ | Hypothesis statement | Introduction |
| √ | Description of study outcomes | Methods |
| √ | Type of exposure or intervention used | Methods |
| √ | Type of study designs used | Methods. Appendix S1. |
| √ | Study population | Methods. Appendices S1 and S2. |
| **Reporting of search strategy should include** | |  |
| √ | Qualifications of searchers | Not applicable. This is a collaborative meta-analysis of individual-level data and thus not based on a search of published literature. |
| √ | Search strategy, including time period included in the synthesis and keywords | Not applicable: see above. |
| √ | Databases and registries searched | Not applicable: see above. |
| √ | Search software used, name and version, including special features | Not applicable: see above. |
| √ | Use of hand searching | Not applicable: see above. |
| √ | List of citations located and those excluded, including justifications | Not applicable: see above. |
| √ | Method of addressing articles published in languages other than English | Not applicable: see above. |
| √ | Method of handling abstracts and unpublished studies | Not applicable: see above. |
| √ | Description of any contact with authors | Not applicable: see above. |
| **Reporting of methods should include** | |  |
| √ | Description of relevance or appropriateness of studies assembled for assessing the hypothesis to be tested | Methods. Appendix S1. |
| √ | Rationale for the selection and coding of data | Methods. Appendix S1. |
| √ | Assessment of confounding | Methods. |
| √ | Assessment of study quality, including blinding of quality assessors; stratification or regression on possible predictors of study results | Methods |
| √ | Assessment of heterogeneity | Methods |
| √ | Description of statistical methods in sufficient detail to be replicated | Methods |
| √ | Provision of appropriate tables and graphics | Table 1, Figures 1 and 2, Appendix S2. |
| **Reporting of results should include** | |  |
| √ | Graph summarizing individual study estimates and overall estimate | Figures 1 and 2. |
| √ | Table giving descriptive information for each study included | Table 1. |
| √ | Results of sensitivity testing | Results. Appendix S2. |
| √ | Indication of statistical uncertainty of findings | 95% confidence intervals have been reported for all effect estimates throughout the manuscript and uncertainty discussed in the Discussion-section. |
| **Reporting of discussion should include** | |  |
| √ | Quantitative assessment of bias | This is a collaborative meta-analysis, so publication bias is not applicable. As we used both published and unpublished harmonised individual-level data, our findings are likely to be more robust to publication bias than findings of literature-based meta-analyses.  Possible other biases: Discussion.  Sensitivity analyses: Results and Appendix S2. |
| √ | Justification for exclusion | As this is a collaborative meta-analysis, all studies in which the investigators were willing to participate were included. |
| √ | Assessment of quality of included studies | Discussion |
| **Reporting of conclusions should include** | |  |
| √ | Consideration of alternative explanations for observed results | Discussion |
| √ | Generalization of the conclusions | Discussion |
| √ | Guidelines for future research | Discussion |
| √ | Disclosure of funding source | Online submission system |

**MOOSE flow chart:** We have not included a flow chart of the literature search process because ours is a collaborative meta-analysis of individual-level data and thus not based on a search of published literature.
